# Quantitative PCR Assays for Detecting Loach Minnow (*Rhinichthys cobitis*) and Spikedace (*Meda fulgida*) in the Southwestern United States

**DOI:** 10.1371/journal.pone.0162200

**Published:** 2016-09-01

**Authors:** Joseph C. Dysthe, Kellie J. Carim, Yvette M. Paroz, Kevin S. McKelvey, Michael K. Young, Michael K. Schwartz

**Affiliations:** 1 United States Department of Agriculture, Forest Service, National Genomics Center for Wildlife and Fish Conservation, Rocky Mountain Research Station, Missoula, MT, United States of America; 2 United States Department of Agriculture, Forest Service, Southwestern Region, Albuquerque, NM, United States of America; University of York, UNITED KINGDOM

## Abstract

Loach minnow (*Rhinichthys cobitis*) and spikedace (*Meda fulgida*) are legally protected with the status of Endangered under the U.S. Endangered Species Act and are endemic to the Gila River basin of Arizona and New Mexico. Efficient and sensitive methods for monitoring these species’ distributions are critical for prioritizing conservation efforts. We developed quantitative PCR assays for detecting loach minnow and spikedace DNA in environmental samples. Each assay reliably detected low concentrations of target DNA without detection of non-target species, including other cyprinid fishes with which they co-occur.

## Introduction

Loach minnow (*Rhinichthys cobitis*) and spikedace (*Meda fulgida*) are cyprinid fishes that were historically widespread throughout the Gila River basin in New Mexico and Arizona [1]. Their populations have declined over the past century due to water development causing altered flow regimes, habitat destruction, and population fragmentation [2–4]. Invasions by nonnative species have exacerbated these declines, and both species are now found in a fraction of their historical ranges [1, 5–6]. In 1986, both species were federally listed as threatened under the US Endangered Species Act [7–8]; their status was elevated to endangered in 2012 [9].

To prioritize conservation efforts for these species, reliable methods to assess their presence and distribution are needed. Traditional sampling methods include electrofishing, seine fishing, and dip netting [4–5]. However, these species are elusive and often occur at low densities in desert streams that undergo periodic flash floods [3, 10]. As a result, traditional sampling methods may not be reliable for understanding patterns of habitat occupancy and the current range of these species.

Environmental DNA (eDNA) is emerging as an efficient and useful tool for detecting rare or invasive aquatic species [11–12] and delimiting distributions of rare species [13]. Combined with quantitative PCR (qPCR) using TaqMan^™^ assays with minor groove binding probes (TaqMan MGB; Applied Biosystems—Life Technologies Corporation), eDNA analysis has proven effective in detecting low concentrations of targeted DNA, and is more sensitive than traditional PCR methods [14]. Here, we describe separate qPCR assays to detect loach minnow and spikedace DNA in environmental samples.

## Methods and Results

To develop qPCR assays specific to loach minnow and spikedace, we compiled GenBank DNA sequences of the cytochrome *b* (cyt*b*) mitochondrial gene for both species as well as eleven non-target fish species commonly found in the same region (Table 1). We used the *DECIPHER* package [15] in *R* v. 3.0.1 [16] to screen sequences *in silico* and obtain primers unique to each target species (Table 2). We aligned and visually compared the primers with sequences of each target and non-target species in MEGA 6.0 [17] and optimized annealing temperatures (Tm) by adjusting primer lengths in Primer Express 3.0.1 (Life Technologies; Table 2). The primers amplify a 164- and 83-base-pair fragment of the cyt*b* gene in loach minnow and spikedace, respectively. Using the MEGA sequence alignments, we designed TaqMan MGB probes (Applied Biosystems; Table 2) with 6-carboxyfluorescein (FAM)-labeled 5’ ends and minor groove binding, non-fluorescent quenchers (MGB-NFQ) for both species by visually identifying species-specific regions. We assessed annealing temperature of the probe for each assay in Primer Express 3.0.1 (Life Technologies; Table 2) and screened each assay for secondary structures using IDT OligoAnalyzer (https://www.idtdna.com/calc/analyzer). Each primer-probe set identically matched all available sequences of each target species, and there was a minimum of two mismatches between the probe and the most closely related non-target species in both sets (Table 1).

**Table 1:** Species, sample size, and GenBank accession number for DNA sequences used for *in silico* marker development.

Common name	Species name	Sequences	Mismatches with loach minnow probe	Mismatches with spikedace probe	GenBank accession number
Loach minnow	*Rhinichthys cobitis*	4	0	4	JX442985 –JX442987; KC763682
Spikedace	*Meda fulgida*	4	5	0	AF452093 –AF452094; JX443054 –JX443055
Colorado pikeminnow	*Ptychocheilus lucius*	2	4	4	JX443071 –JX443072
Common carp	*Cyprinus carpio*	2	4	6	KF574485; KF574490
Desert pupfish	*Cyprinodon macularius*	1	6	5	AY902103
Desert sucker	*Catostomus clarkii*	2	5	4	JX488779; KJ441261
Fathead minnow	*Pimephales promela*	2	5	2	GQ184520; GQ275159
Gila chub	*Gila intermedia*	2	3	6	JX443036; KF514914
Gila topminnow	*Poeciliopsis occidentalis*	2	8	6	AF412140; AF412144
Headwater chub	*Gila nigra*	2	3	6	JX443028; KF514210
Longfin dace	*Agosia chrysogaster*	2	5	5	DQ324093; JX443014
Razorback sucker	*Xyrauchen texanus*	2	5	6	AF454869; JX488824
Red shiner	*Cyprinella lutrensis*	1	2	3	KR061540
Roundtail chub	*Gila robusta*	2	3	5	JX443035; KF514254
Sonora sucker	*Catostomus insignis*	2	5	5	JX488786; KJ441283
Speckled dace	*Rhinichthys osculus*	4	5	3	DQ990313 –DQ990316

**Table 2:** Primers and probes to detect loach minnow and spikedace using qPCR.

Assay component	Sequence (5’-3’)	Tm (°C)	Final concentration (nM)
Loach minnow forward primer	CTTACCCAGTTCCTATTTTGGACACT	59	100
Loach minnow reverse primer	ATTCTCTATCCATCCTGCGAGC	58.4	300
Loach minnow probe	FAM-TGGCGGATATACTCATCCT-MGBNFQ	70	250
Spikedace forward primer	GTAGCGGACGTACTTATTCTTACCTGA	59.2	100
Spikedace reverse primer	AAAGTATAACAGGGATGCGATTTGTC	59.7	600
Spikedace probe	FAM-GAACACCCATATGTCGC-MGBNFQ	69	250

To test the specificity of the loach minnow assay, we screened DNA extracted from 13 loach minnow tissues from 4 locations in Arizona and 19 additional non-target species (Table 3). Similarly, for the spikedace assay we screened DNA extracted from 9 spikedace tissues from 3 locations in Arizona and 19 additional species (Table 3). DNA from tissue was extracted with the DNeasy Tissue and Blood Kit (Qiagen, Inc.) using the manufacturer’s protocol. All tissues were collected under state and federal permits issued by the U. S. Fish and Wildlife Service (Arizona Game and Fish’s 10(a)1(a) permit: NATIVE ENDANGERED AND THREATENED SPECIES RECOVERY—E & T #TE821577-5), the Arizona Game and Fish Department permit #SP746929, and the New Mexico Department of Game and Fish permit #1899. Tissues were obtained by removing a small fin clip and immediately releasing the fish at the point of capture. The process required minimal handling and was performed quickly, minimizing stress on the fish. We used the extracted tissue DNA to test *in vitro* each qPCR assay with a StepOne Plus Real-time PCR Instrument (Life Technologies) in 15-μl reactions containing 7.5 μl Environmental Master Mix 2.0 (Life Technologies), 900 nM forward primer, 900 nM reverse primer, 250 nM probe, 4 μl DNA template (~0.12–0.88 ng), and 2.75 μl deionized water. Thermocycler conditions included 95°C for 10 min followed by 45 cycles of denaturation at 95°C for 15 s and annealing at 60°C for 1 min. Due to the susceptibility of qPCR to contamination, all qPCR tests were set up inside of a UV hood where consumables and pipettes were irradiated with UV light for 1 h prior to each test. Each test included a no-template control (NTC) with distilled water used in place of DNA template. For both assays, DNA from all target species was detected, and there was no detection of DNA from non-target species or the NTC.

**Table 3:** List of species used for *in vitro* screening of the primers and probe. Origin refers to the waterbody for loach minnow and spikedace samples. For all other samples, origin is listed as state.

Common name	Species name	Loach minnow probe testing sample size	Spikedace probe testing sample size	Origin
Loach minnow	*Rhinichthys cobitis*	3	1	Aravaipa Creek, AZ
		3	1	Blue River, AZ
		4	2	Gila Forks, AZ
		3	1	San Francisco River, AZ
Spikedace	*Meda fulgida*	1	3	Aravaipa Creek, AZ
		1	3	Gila Forks, AZ
		1	3	Gila River, AZ
Apache trout	*Oncorhynchus apache*	1	1	NM
Brook trout	*Salvelinus fontinalis*	1	1	ID
Brown trout	*Salmo trutta*	1	1	NM
Channel catfish	*Ictalurus punctatus*	1	1	MT
Colorado pikeminnow	*Ptychocheilus lucius*	1	1	UT
Common carp	*Cyprinus carpio*	1	1	MT
Desert sucker	*Catostomus clarkii*	1	1	NM
Fathead minnow	*Pimephales promelas*	3	3	MT, NM
Flathead chub	*Platygobio gracilis*	1	1	MT
Gila trout	*Oncorhynchus gilae*	1	1	NM
Longfin dace	*Agosia chrysogaster*	1	1	NM
Longnose dace	*Rhinichthys cataractae*	1	1	MT
Razorback sucker	*Xyrauchen texanus*	2	2	AZ, UT
Red shiner	*Cyprinella lutrensis*	1	1	NM
River carpsucker	*Carpiodes carpio*	1	1	MT
Sand shiner	*Notropis stramineus*	1	1	MT
Sonora sucker	*Catostomus insignis*	1	1	NM
Speckled dace	*Rhinichthys osculus*	4	3	AZ, NM

We optimized assay concentrations following methods outlined in Wilcox et al. [18] (Table 2). Using the optimized assay concentrations and cycling conditions above, we tested assay sensitivity by performing standard curve experiments created from target qPCR product. For each assay, qPCR product was purified using PureLink^™^ PCR Micro Kit (Invitrogen) and quantified on a Qubit 2.0 fluorometer (ThermoFisher Scientific). From this stock, we prepared a six-level standard curve dilution series (6 250, 1 250, 250, 50, 10, and 2 copies per 4 μl) in sterile TE. We ran six replicates of each dilution resulting in an amplification efficiency of 100.3% (standard curve y-intercept = 40.229, *r*^2^ = 0.99) and 91.7% (standard curve y-intercept = 40.948, *r*^2^ = 0.993) for the loach minnow and spikedace assays respectively. The limit of detection (lowest concentration with >95% amplification success [19]) of each assay was 10 mtDNA copies/rxn with successful detection of target DNA in all six replicates; however, each assay also detected target DNA in five out of six replicates at 2 mtDNA copies/rxn (83.3% detection success).

Finally, for each assay we screened eDNA samples collected from two southwestern U.S. sites for which the cyprinid community assemblage was known from previous electrofishing surveys (Table 4). We also screened five eDNA samples filtered from hatchery tanks containing known fishes (Table 4). Environmental DNA was collected from 5-l water samples at field sites and 1-l samples at hatchery sites following methods described in Carim et al. [20], and extracted with the DNeasy Tissue and Blood Kit (Qiagen, Inc.) using a modified protocol [21]. Using the optimized PCR conditions above, we analyzed these environmental samples with each assay and screened for PCR inhibition with an internal positive control. As expected, the assays detected loach minnow and spikedace eDNA in all samples collected where these species were known to be present, but not in any of the samples collected where these species are suspected absent.

**Table 4:** Collection and species assemblage information for eDNA samples used to test the loach minnow and spikedace qPCR assays.

Waterbody (State)	Latitude	Longitude	Collection date	Cyprinid species present*	Loach minnow expected/ detected?	Spikedace expected/ detected?
Aravaipa Creek (AZ)	32.897797	-110.441802	3/13/2016	DSR, LFD, LMW, RSR, RTC, SPD, SKD, SSR	Y/Y	Y/Y
Beaver Creek (AZ)	34.668761	-111.714222	3/20/2016	DSR, LFD, RTC	N/N	N/N
AZ Game & Fish—Aquatic Research and Conservation Center- Tank Sample (AZ)	34.764735	-111.894515	3/11/2016	BTC, CPM, DPF, HBC, LMW, LFD, RBS, RSR, RTC, SPD, SKD, WNF	Y/Y	Y/Y
AZ Game & Fish—Aquatic Research and Conservation Center- Tank Sample (AZ)				SKD	N/N	Y/Y
AZ Game & Fish—Aquatic Research and Conservation Center- Tank Sample (AZ)				LMW	Y/Y	N/N
AZ Game & Fish—Aquatic Research and Conservation Center- Tank Sample (AZ)				RTC	N/N	N/N
AZ Game & Fish—Aquatic Research and Conservation Center- Tank Sample (AZ)				WNF	N/N	N/N

*BTC = bonytail chub (*Gila elegans*); CPM = Colorado pikeminnow; DPF = desert pupfish; DSR = desert sucker; HBC = humpback chub (*Gila cypha*); LFD = longfin dace; LMW = loach minnow; RBS = razorback sucker; RSR = red shiner; RTC = roundtail chub; SPD = speckled dace; SKD = spikedace; SSR = Sonora sucker; WNF = woundfin (*Plagopterus argentissimus*)

## Discussion

The qPCR assays we describe here are species specific and highly sensitive, consistently detecting low quantities of loach minnow and spikedace DNA. With these assays, biologists will be able to rapidly and reliably assess population distributions of these threatened fishes. Furthermore, the nature of this method allows for easily repeatable sampling and analysis over time [13], providing valuable information on the temporal dynamics of loach minnow and spikedace populations. Ultimately, this information will help managers prioritize conservation efforts for these species in the southwestern U.S.

## References

[1] Paroz YM, Propst DL. Distribution of spikedace, loach minnow, and chub species in the Gila River Basin, New Mexico 1908–2007. Report to U.S. Fish and Wildlife Service and U.S. Bureau of Reclamation. New Mexico Department of Game and Fish, Santa Fe. July 2007.

[2] USFWS (United States Fish and Wildlife Service). Loach minnow recovery plan. Albuquerque, New Mexico 1990; 38 pp.

[3] USFWS. Spikedace recovery plan. Albuquerque, New Mexico 1990; 38 pp.

[4] Propst DL, Bestgen KR. Habitat and biology of the loach minnow, *Tiaroga cobitis*, in New Mexico. Copeia. 1991; 1991(1):29–38. 10.2307/1446245

[5] Barber WE, Williams DC, Minckley WL. Biology of the Gila spikedace, *Meda fulgida*, in Arizona. Copeia. 1970; 1: 9–18.

[6] Propst DL, Bestgen KR, Painter CW. Distribution, status, biology, and conservation of the loach minnow (*Tiaroga cobitis*) in New Mexico Endangered Species Report No. 17. U.S. Fish and Wildlife Service, Albuquerque, NM 3 1988.

[7] USFWS. Endangered and threatened wildlife and plants: determination of threatened status for the loach minnow. Federal Register 1986; 51: 39468–39478.

[8] USFWS. Endangered and threatened wildlife and plants: determination of threatened status for the spikedace. Federal Register 1986; 51: 23769–23781.

[9] USFWS. Endangered and threatened wildlife and plants: endangered status and designations of critical habitat for spikedace and loach minnow. Federal Register 2012; 77: 10810–10932.

[10] Marsh PC, Bagley BE, Knowles GW, Schiffmiller G, Sowka PA. New and rediscovered populations of loach minnow, *Tiaroga cobitis* (Cyprinidae), in Arizona. Southwest Nat. 2003; 48(4):666–669.

[11] Dejean T, Valentini A, Miquel C, Taberlet P, Bellemain E, Miaud C Improved detection of an alien invasive species through environmental DNA barcoding: the example of the American bullfrog *Lithobates catesbeianus*. J Appl Ecol. 2012; 49: 953–959. 10.1111/j.1365-2664.2012.02171.x

[12] Siggsgaard AA, Carl H, Moller P, Thomsen PF. Monitoring near-extinct of European weather loach in Denmark based on enviornmental DNA from water samples. Biol Conserv. 2015; 183: 46–52. 10.1016/j.biocon.2014.11.023

[13] McKelvey KS, Young MK, Knotek EL, Carim KJ, Wilcox TM, Padgett-Stewart TM, et al Sampling large geographic areas for rare species using environmental DNA (eDNA): a study of bull trout *Salvelinus confluentus* occupancy in western Montana. J Fish Biol. 2016; 88: 1215–1222. 10.1111/jfb.12863 26762274

[14] Wilcox TM, McKelvey KS, Young MK, Jane SF, Lowe WH, Whiteley AR, et al Robust detection of rare species using environmental DNA: The importance of primer specificity. PLoS ONE. 2013; 8(3): e59520 10.1371/journal.pone.0059520 23555689 PMC3608683

[15] Wright ES, Yilmaz LS, Ram S, Gasser JM, Harrington GW, Noquera DR. Exploiting extension bias in polymerase chain reaction to improve primer specificity in ensembles of nearly identical DNA templates. Environ Microbiol. 2014; 16: 1354–1365. 10.1111/1462-2920.12259 24750536

[16] R Core Development Team. R: A language and environment for statistical computing [Internet]. Vienna, Austria: Foundation for Statistical Computing; 2013 Available: http://www.r-project.org/

[17] Tamura K, Peterson D, Peterson N, Filipski A, Kumar S. MEGA6: molecular evolutionary genetics analysis version 6.0. Mol Biol Evol. 2013; 30: 2725–2729. 10.1093/molbev/mst197 24132122 PMC3840312

[18] Wilcox TM, Carim KJ, McKelvey KS, Young MK, Schwartz MK. The dual challenges of generality and specificity with developing environmental DNA markers for species and subspecies of *Oncorhynchus*. PLoS ONE. 2015; 10(11): e0142008 10.1371/journal.pone.0142008 26536367 PMC4633235

[19] Bustin SA, Benes V, Garson JA, Hellemans J, Huggett J, Kubista M, et al The MIQE guidelines: minimum information for publication of quantitative real-time PCR experiments. Clin Chem. 2009: 55: 611–622. 10.1373/clinchem.2008.112797 19246619

[20] Carim KJ, Padgett-Stewart T, Wilcox TM, Young MK, McKelvey KS, Schwartz MK. Protocol for collecting eDNA samples from streams. USDA Forest Service—National Genomics Center for Wildlife and Fish Conservation. 2015; V2.3 (July 2015). Available: http://www.fs.fed.us/research/genomics-center/docs/edna/edna-protocol.pdf.

[21] Carim KJ, Dysthe JCS, Young MK, Mckelvey KS, Schwartz MK. An environmental DNA assay for detecting Arctic grayling in the upper Missouri River basin, North America. Conserv Genet Resour. 2016 4 01.

